# New severity score of acute respiratory failure

**DOI:** 10.1186/cc11022

**Published:** 2012-03-20

**Authors:** S Allal, A Khedher, I Ben Saida, A Azouzi, A Farjallah, I Chouchen, S Bouchoucha, M Boussarsar

**Affiliations:** 1 CHU Farhat Hached Hospital, Sousse, Tunisia

## Introduction

Acute respiratory failure (ARF), a common syndrome, is still poorly clinically appreciated. Literature review reports only a few attempts in neonatology (Silverman score) and in adults (Patrick score [1]) constructed by authors in 1996 for scientific research purposes. Both scores have never been validated. Instead, clinicians developed specific scores. We constructed a new respiratory failure score, organized in a trimodal manner (Table 1). Items were selected on the basis of pathophysiological and clinical expertise. Particular attention was paid to formulation and scaling to make the score both simple, noninvasive, inexpensive, didactic, and with interesting clinimetric properties. The objective of this study is to validate this score already in use for several years in our ICU.

**Table 1:** Score of respiratory failure

Grade	Respiratory rate	Accessory muscle use	Hypoxemia
I	<30	Intercostal	Normal
II	30 to 40	Supraclavicular and/or suprasternal	Cyanosis
III	>40	Thoraco-abdominal	Circulatory and/or
		swing/nasal flaring	consciousness disorders
IV	Gasp	Exhaustion/ventilatory arrest	Cardio-circulatory arrest

## Methods

A prospective study among 70 patients with ARF on previously healthy lungs. ARF was rated in a randomized blinded manner respectively by residents and seniors. An inter-rater reliability analysis using the kappa statistic was performed to determine consistency among raters. Clinimetric properties were assessed by examining the prognostic prediction by the ROC curve using a composite gold standard (PaO_2_/FiO_2 _<250 and/or ventilatory support).

## Results

The inter-rater reliability for the raters was found to be κ = 0.82 (*P *< 0.001), indicating an almost perfect agreement [2]. The area under the ROC curve was revealed very interesting (AUC = 0.88) indicating an excellent prognostic predictive power.

## Conclusion

This new and validated score could drive some advantages in daily practice, allowing accurate assessment of ARF severity, more objective monitoring of patients and easier communication between care providers. It may accurately guide oxygen supplementation and ventilatory support and afford accurate monitoring of patho-physiological and etiological treatment of ARF. It could be a valuable tool in randomized clinical trials or physiological studies evaluating treatments in ARF. Finally it could be used as an educational tool.
